# Diagnostic performance of the LRINEC score for diagnosing necrotizing fasciitis in a Latin American population

**DOI:** 10.15649/cuidarte.5497

**Published:** 2026-09-11

**Authors:** Cristian David Arévalo Mojica, Laura Cristina Nocua-Báez, Paula Andrea González Caicedo, Sugeich del Mar Meléndez Rhenals, Carlos Humberto Saavedra Trujillo

**Affiliations:** 1 Department of Internal Medicine, Faculty of Medicine, Universidad Nacional de Colombia. Hospital Universitario Nacional de Colombia. Bogotá. Colombia. E-mail: cdarevalom@unal.edu.co Universidad Nacional de Colombia Bogotá Colombia cdarevalom@unal.edu.co; 2 Department of Internal Medicine, Faculty of Medicine, Universidad Nacional de Colombia. Clínica Universitaria Colombia. Bogotá. Colombia. E-mail: lcnocuab@unal.edu.co Universidad Nacional de Colombia Bogotá Colombia lcnocuab@unal.edu.co; 3 Hospital Universitario Nacional de Colombia. Bogotá. Colombia. E-mail: metodologoinvestigacion3@hun.edu.co Hospital Universitario Nacional de Colombia Bogotá Colombia metodologoinvestigacion3@hun.edu.co; 4 Department of Internal Medicine, Faculty of Medicine, Universidad Nacional de Colombia. Hospital Universitario Nacional de Colombia. Bogotá. Colombia. E-mail: summelendezrh@unal.edu.co Universidad Nacional de Colombia Bogotá Colombia summelendezrh@unal.edu.co; 5 Department of Internal Medicine, Faculty of Medicine, Universidad Nacional de Colombia. Hospital Universitario Nacional de Colombia. Clínica Universitaria Colombia. Bogotá. Colombia. E-mail: chsaavedrat@unal.edu.co Universidad Nacional de Colombia Bogotá Colombia chsaavedrat@unal.edu.co

**Keywords:** Fasciitis, Necrotizing, Cellulitis, Soft Tissue Infections, Forecasting, Sensitivity and Specificity, Fascitis Necrotizante, Celulitis, Infecciones de los Tejidos Blandos, Predicción, Exactitud de las Pruebas Diagnósticas, Fasciite Necrosante, Celulite, Infecções dos Tecidos, Moles Predição, Acurácia dos Testes Diagnósticos

## Abstract

**Introduction:**

The Laboratory Risk Indicator for Necrotizing Fasciitis (LRINEC) score was developed to estimate the likelihood of necrotizing fasciitis (NF); however, its diagnostic performance varies across populations, and evidence from Latin America remains limited.

**Objective:**

To assess the diagnostic performance of the LRINEC score in a Colombian population with skin and soft tissue infections.

**Materials and Methods:**

A retrospective cohort study was conducted among patients hospitalized at two tertiary care institutions in Colombia between 2016 and 2021. Patients with signs of systemic inflammatory response were considered suspected cases of NF. The diagnosis was confirmed or ruled out based on intraoperative or histopathologic findings. The LRINEC score was calculated, and its diagnostic performance was evaluated using sensitivity, specificity, and receiver operating characteristic (ROC) curves.

**Results:**

Among 1.546 medical records, 29 confirmed cases of NF and 106 suspected cases in which NF was ruled out were identified. The mean LRINEC score among patients with NF was 6 points. A cutoff score of ≥6 yielded a sensitivity of 62.07%, a specificity of 71.70%, and an area under the ROC curve (AUC) of 0.67. At a cutoff score of ≥8, sensitivity was 34.48%, and specificity was 89.62%.

**Discussion:**

These findings indicate moderate diagnostic performance, similar to that reported in other retrospective studies conducted in Latin American populations, with greater utility for ruling out NF than for ruling it in.

**Conclusion:**

The LRINEC score showed limited utility as a standalone diagnostic tool. A score <6 had a high negative predictive value and should always be interpreted in conjunction with clinical judgment.

## Resumen

**Introducción:**

La escala LRINEC (Laboratory Risk Indicator for Necrotizing Fasciitis Score) busca predecir el riesgo de fascitis necrosante (FN), sin embargo, su rendimiento es variable entre diferentes poblaciones y existe poca información en Latinoamérica.

**Objetivo:**

Evaluar el rendimiento diagnóstico de LRINEC en una población colombiana con infección de piel y tejidos blandos.

**Materiales y métodos:**

Estudio de cohorte retrospectiva de pacientes hospitalizados en dos instituciones colombianas de alta complejidad entre 2016 y 2021. Se consideraron sospechosos de FN aquellos con signos de respuesta inflamatoria sistémica. El diagnóstico se confirmó o descartó mediante descripción quirúrgica o patología. Se calculó el puntaje LRINEC y se evaluó su rendimiento diagnóstico mediante sensibilidad, especificidad y curvas ROC.

**Resultados:**

De 1546 registros clínicos, se identificaron 29 casos confirmados de FN y 106 casos sospechosos con FN descartada. El puntaje promedio de LRINEC fue de 6 puntos en el grupo con FN. Un puntaje ≥6 presentó sensibilidad de 62,07%, especificidad de 71,70% y área bajo la curva ROC de 0,67. Para un puntaje ≥8, la sensibilidad fue de 34,48% y la especificidad de 89,62%.

**Discusión:**

Los hallazgos muestran un rendimiento diagnóstico moderado, similar al reportado en otros estudios retrospectivos latinoamericanos, con mayor utilidad para descartar FN que para confirmarla.

**Conclusiones:**

La escala LRINEC mostró utilidad limitada como herramienta diagnóstica aislada; un puntaje <6 presentó un alto valor predictivo negativo y debe interpretarse junto con el juicio clínico.

## Resumo

**Introdução:**

A escala LRINEC (Laboratory Risk Indicator for Necrotizing Fasciitis Score) visa predizer o risco de fasciite necrotizante (FN); entretanto, seu desempenho varia entre diferentes populações, e há pouca informação disponível na América Latina.

**Objetivo:**

Avaliar o desempenho diagnóstico da LRINEC em uma população colombiana com infecções de pele e tecidos moles.

**Materiais e métodos:**

Estudo de coorte retrospectivo de pacientes hospitalizados em duas instituições colombianas de alta complexidade entre 2016 e 2021. Pacientes com sinais de resposta inflamatória sistêmica foram considerados suspeitos de FN. O diagnóstico foi confirmado ou descartado por meio de descrição cirúrgica ou histopatológica. O escore LRINEC foi calculado e seu desempenho diagnóstico foi avaliado utilizando sensibilidade, especificidade e curvas ROC.

**Resultados:**

De 1546 prontuários clínicos, foram identificados 29 casos confirmados de FN e 106 casos suspeitos com FN descartada. A pontuação média do LRINEC foi de 6 pontos no grupo com neutropenia febril (NF). Uma pontuação ≥6 apresentou sensibilidade de 62,07%, especificidade de 71,70% e área sob a curva ROC de 0,67. Para uma pontuação ≥8, a sensibilidade foi de 34,48% e a especificidade de 89,62%.

**Discussão:**

Os achados demonstram desempenho diagnóstico moderado, semelhante ao relatado em outros estudos retrospectivos latino-americanos, com maior utilidade para descartar NF do que para confirmá-la.

**Conclusões:**

A escala LRINEC apresentou utilidade limitada como ferramenta diagnóstica isolada; uma pontuação <6 apresentou alto valor preditivo negativo e deve ser interpretada em conjunto com o julgamento clínico.

## Introduction

Necrotizing fasciitis (NF) is a skin and soft tissue infection (SSTI) characterized by progressive destruction of the muscular fascia and adjacent subcutaneous adipose tissue^1^. It is potentially life-threatening and associated with high morbidity, with a rapidly progressive and severe course that can lead to sepsis and multiorgan dysfunction^2^. Timely identification of NF is associated with improved clinical outcomes^3^. However, timely diagnosis remains clinically challenging because its signs and symptoms often overlap with those of other non-necrotizing SSTIs and because clinical findings specific to NF often emerge late^4^.

Diagnostic tools are intended to support diagnosis or raise clinical suspicion of the condition in order to guide patients toward timely surgical management, thereby improving survival and reducing morbidity^5^. One such instrument is the Laboratory Risk Indicator for Necrotizing Fasciitis (LRINEC) score, developed in 2004 to identify patients at increased risk of NF^6^. The score is calculated using laboratory parameters (serum sodium, glucose, and creatinine; C-reactive protein level; leukocyte count; and hemoglobin level). The total score ranges from 0 to 13. Scores ≥6 are associated with a high risk of NF (positive predictive value of 92% and negative predictive value of 96% in the original study), while scores ≥8 indicate a very high risk of NF, with a positive predictive value of 93.4%^6^.

The external validity and diagnostic performance of the LRINEC score have varied across the different populations evaluated; however, its high specificity and association with increased morbidity and prolonged hospital stays have been highlighted^7^,^8^. Nevertheless, these studies did not include Latin American populations. More recent publications, such as the retrospective study conducted in 2025 by Norimatsu et al. to evaluate the diagnostic performance of the LRINEC score in a Japanese population, reported a sensitivity of 68.9% and a specificity of 96%^9^. Likewise, a 2025 retrospective study conducted in a Hispanic population in Spain found that an LRINEC score > 6 may facilitate early diagnosis and be associated with the severity of the septic process; however, the study did not report the score’s predictive values^10^. In Latin America, available evidence remains scarce. A study conducted in Cuba in 2018 demonstrated the utility of the LRINEC score for the diagnosis of NF, reporting an area under the ROC curve of 0.607 (95% CI: 0.47–0.73), with a sensitivity of 66% and specificity of 75%^11^. However, the diagnostic performance of the LRINEC score in the Colombian population remains unknown. Therefore, this study aimed to evaluate the diagnostic performance of the LRINEC score in a Colombian population with skin and soft tissue infections.

## Materials and Methods

**Study design**

A retrospective cohort study was conducted among patients hospitalized at two tertiary care university hospitals in Bogotá, Colombia (Hospital Universitario Nacional de Colombia and Clínica Universitaria Colombia). Patients diagnosed with skin and soft tissue infections (SSTIs), including cellulitis, cutaneous abscesses, and localized skin and subcutaneous tissue infections, between January 2016 and December 2021 were included.

**Population and sample**

All electronic medical records of hospitalized patients with SSTI diagnoses coded according to the International Classification of Diseases, 10th Revision (ICD-10), during the study period were identified. Records of outpatient care, diagnoses other than SSTIs, duplicate medical records, and records of patients younger than 18 years of age were excluded. Records were also excluded if they contained incomplete information, lacked data required to calculate the LRINEC score, or did not contain sufficient information to confirm or rule out NF.

**Data collection**

Data were collected through a systematic review of institutional electronic medical records. Demographic, clinical, and laboratory variables recorded during the first 48 hours of hospitalization were extracted using a structured approach. The data were recorded in a standardized data collection matrix designed for the study.

**Definition of suspected cases and follow-up**

Patients were considered suspected cases of NF if they exhibited at least two clinical signs of systemic inflammatory response within the first 48 hours of hospitalization: tachycardia (heart rate >90 beats/min), tachypnea (respiratory rate >20 breaths/min), or fever (body temperature >38°C). Suspected cases were followed throughout hospitalization until NF was confirmed or ruled out using the reference standard based on intraoperative or histopathologic findings. Laboratory test results obtained within the first 48 hours of admission were used to calculate the LRINEC score, and the diagnostic performance and operational characteristics of the score were assessed.

**Bias control**

To minimize selection bias, all eligible patients hospitalized during the study period were included, and predefined exclusion criteria were applied consistently. To minimize information bias, only variables recorded within the first 48 hours of hospitalization were analyzed, and laboratory results were cross-checked against two sources in the medical record. Records with insufficient data were also excluded. To address potential confounding, between-group comparisons were performed using statistical tests appropriate for the distribution of the data, and conclusions were limited to the diagnostic performance of the LRINEC score, without making causal inferences.

**Statistical analysis**

The sociodemographic and clinical characteristics of the study population were summarized. A logistic regression model was fitted to predict the diagnosis of necrotizing fasciitis (NF) and determine the slope of the ROC curve. Confirmed NF was the dependent variable, and the components of the LRINEC score were the independent variables. Peduzzi et al.^12^ recommend including 5 to 10 outcome events per predictor variable in regression models. Given that the LRINEC score comprises six variables, a minimum of 30 NF events was established for this study. Univariate and bivariate analyses were performed using graphical methods and appropriate summary statistics, including measures of central tendency, dispersion, and position, as appropriate for the type of variable. Diagnostic performance was estimated in terms of sensitivity, specificity, positive and negative predictive values, likelihood ratios (LR+ and LR−), and the area under the ROC curve, thereby providing an overall assessment of the operating characteristics of the LRINEC score.

All statistical analyses were performed using Stata version 17 and R version 4.1. The data used in this study are publicly available in Mendeley Data^13^.

**Ethical considerations**

This study adhered to Good Clinical Practice guidelines for research involving human subjects and was conducted in accordance with current Colombian regulations established under Resolution 8430 of 1993, Article 11^14^. The study protocol was approved by the Research Ethics Committees of Hospital Universitario Nacional de Colombia and Clínica Universitaria Colombia (approval references Minutes CEI-HUN-ACTA-2023-12 and Minutes 020-23 dated May 30, 2023, respectively).

## Results

A total of 2,147 medical records of hospitalized patients with SSTI diagnoses coded according to the International Classification of Diseases, 10th Revision (ICD-10), were identified during the study period. Of these, 601 records were excluded because they corresponded to outpatient care, were duplicates, involved diagnoses other than SSTIs, or met the age exclusion criteria. The remaining 1,546 medical records were reviewed to determine whether patients met the criteria for suspected NF and whether the diagnosis was confirmed using the reference standard, based on intraoperative or histopathologic findings.

A total of 256 suspected cases of NF were identified. Of these, NF was confirmed in 32 cases using the reference standard, ruled out in 204 cases, and 20 cases were excluded because of an additional infectious focus. Of the 1,546 SSTI cases, 2.06% (n=32) were diagnosed with NF, whereas 12.50% of patients with suspected NF (n=256) had confirmed NF. No confirmed NF cases were identified among the 1,290 SSTI patients who did not meet the criteria for suspected NF based on intraoperative findings.

Overall, 29 confirmed NF cases and 106 suspected cases in which NF was ruled out based on the reference standard were included in the statistical analysis. Cases with incomplete laboratory data were excluded from the analysis. Figure 1 presents the cohort selection flowchart.

**Figure 1:**
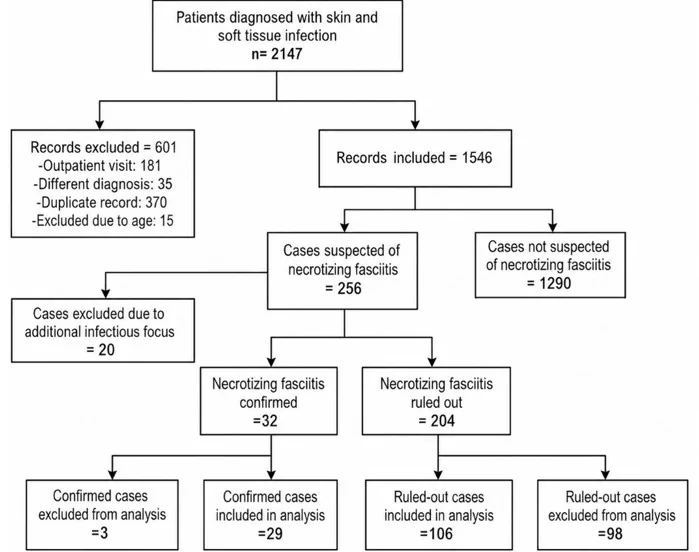
Cohort selection flowchart

**Sociodemographic Characteristics**

The median age at diagnosis was 56 years among patients with confirmed NF and 54 years among those in whom NF was ruled out. Among confirmed NF cases (n=29), a higher proportion of male patients was observed (55.22% [n=16]). Most cases occurred in patients enrolled in the contributory health insurance scheme (86.65% [n=25]), and all were Colombian nationals see Table 1.

**Table 1:** Sociodemographic characteristics

Characteristic	Confirmed NF (n=29) % (n)	NF Ruled Out (n=106) % (n)	Total (n=135) % (n)	p-value
Age, median [IQR], years	56 [23;69]	54 [34;67]	56 [33;67]	0.810
Sex				0.890
Male	55.22 (16 )	53.77 (57)	54.07 (73)	
Female	44.82 (13)	46.22 (49)	45.92 (62)	
Health insurance scheme				0.057
Contributory	86.20 (25)	97.16 (103)	94.81 (128)	
Special	6.89 (2)	0.94 (1)	2.22 (3)	
Other	6.89 (2)	1.88 (2)	2.96 (4)	
Nationality				1.00
Colombian national	100 (29)	97.16 (103)	97.70 (132)	
Foreign national	0 (0)	2.83 (3)	2.22 (3)	

IQR: Interquartile range; NF: Necrotizing fasciitis

**Clinical characteristics**

Among confirmed NF cases (n=29), higher proportions of comorbidities were observed, including hypertension (41.37%), diabetes mellitus (51.72%), and chronic kidney disease (20.70%). However, only diabetes mellitus showed a statistically significant difference compared with the group without NF (p = 0.031), suggesting an association with necrotizing infections. No between-group differences were observed in the frequency of immunosuppression (17.24%).

**Table 2:** Clinical characteristics

Characteristic	Confirmed NF (n=29) % (n)	NF Ruled Out (n=106) % (n)	Total (n=135) % (n)	p-value
Length of hospital stay, median [IQR]	24 [18;52]	8 [4;12]	11.5 [4;16]	< 0.001
Comorbidities				
Hypertension	41.37 (12)	34.90 (37)	36.29 (49)	0.520
Diabetes mellitus	51.72 (15)	30.18 (32)	34.81 (47)	0.031
Chronic kidney disease	20.68 (6)	14.15 (15)	15.55 (21)	0.390
Immunosuppression	17.24 (5)	16.98 (18)	17.03 (23)	0.970
Respiratory rate, median [IQR]	22 [21;24]	22 [21;22]	22 [21;22]	0.990
Heart rate, median [IQR]	100 [95;113]	99 [94;105]	100 [94;106]	0.730
Body temperature Median [IQR]	37 [36.87;38.35]	36.5 [36.07;37.22]	36.55 [36.17;37.50]	0.009
Fever				
Present	37.93 (11)	25.54 (27)	28.14 (38)	0.240
Pain Severity (VAS)				
VAS 0–3: Mild pain	51.72 (15)	84.90 (90)	77.77 (105)	
VAS 4–7: Moderate pain	20.68 (6)	8.49 (9)	11.11 (15)	<0.001
VAS 8–10: Severe pain	27.58 (8)	6.60 (7)	11.11 (15)	

NF: Necrotizing fasciitis; IQR: Interquartile range; VAS: Visual Analog Scale

Regarding clinical presentation, 37.93% of patients with NF had fever. Pain intensity was greater in this group, with moderate pain reported in 20.68% and severe pain in 27.58%. This difference was statistically significant (p < 0.001), highlighting severe pain as an important warning sign of NF.

Hospital length of stay was significantly longer among patients with NF (median 24 days, IQR 18–52; p < 0.001), indicating a more severe clinical course and greater complexity of care. A significant difference in body temperature was also identified (p = 0.009), indicating greater temperature abnormalities among confirmed NF cases.

A high proportion of surgical interventions was observed in the study population (60.00%), and all patients with NF required surgical management. In-hospital mortality was 13.00% in the NF group, whereas no deaths occurred in the group without NF see Table 2.

**Laboratory characteristics (LRINEC components)**

Relevant between-group differences were observed in laboratory parameters. Median C-reactive protein (CRP) levels were higher among NF cases (180 mg/L), although this difference was not statistically significant (p = 0.10). Hemoglobin levels showed a statistically significant difference (p < 0.001), with median values 2 g/dL lower among patients with NF. Likewise, glucose levels were significantly higher in the NF group (median 132 mg/dL; p = 0.033).

Leukocytosis was more pronounced among patients with NF (median leukocyte count of 16,200 cells/µL), and the between-group difference was statistically significant (p = 0.039), supporting its utility as a marker. Conversely, no significant differences were found in serum creatinine (p = 0.24) or serum sodium levels (p = 0.80), suggesting that these parameters did not contribute to discriminating between groups in this population. Detailed results are presented in Table 3.

**Table 3:** Laboratory characteristics

Characteristic	Confirmed NF (n=29) median [IQR]	NF Ruled Out (n=106) median [IQR]	Total (n=135) median [IQR]	p-value
CRP (mg/L)	180 [106;241]	122.5 [37;206]	139[45;213]	0.10
Hemoglobin (g/dL)	11.4 [10;12.6]	12.7 [12;14.8]	12.5 [11.8;14.6]	<0.001
Serum creatinine (mg/dL)	0.87 [0.64;1.33]	0.80 [0.67;1]	0.83 [0.66.1.10]	0.24
Serum sodium (mEq/L)	138 [136;141]	138 [136;140]	138 [136;140]	0.80
Glucose (mg/dL)	132 [113;198]	106 [92;148]	114 [92;154]	0.033
Leukocyte count (cells/µL)	16200 [9175;21898]	11535 [8228;15572]	11810 [8228;16185]	0.039

IQR: Interquartile Range; NF: necrotizing fasciitis; CRP: C-reactive protein (mg/L)

**Evaluation of the LRINEC score**

The mean LRINEC score was 6 points in the NF group and 4 points in the non-NF group. At a cutoff score of ≥6 points, the area under the ROC curve (AUC) was 0.67, with a sensitivity of 62.07% and a specificity of 71.70% Figure 2. Positive and negative predictive values were 37.50% and 87.36%, respectively, with a positive likelihood ratio (LR+) of 2.193 and a negative likelihood ratio (LR−) of 0.529. At an LRINEC cutoff score of ≥8 points, the AUC was 0.6205, with a sensitivity of 34.48% and a specificity of 89.62% Figure 2. Positive and negative predictive values were 47.62% and 83.30%, respectively, with an LR+ of 3.32 and an LR− of 0.73. Based on the AUC and likelihood ratio values, the LRINEC score showed limited discriminatory ability in the study population. Although the score may serve as a complementary diagnostic tool, it is insufficient as a stand-alone diagnostic criterion.

**Figure 2:**
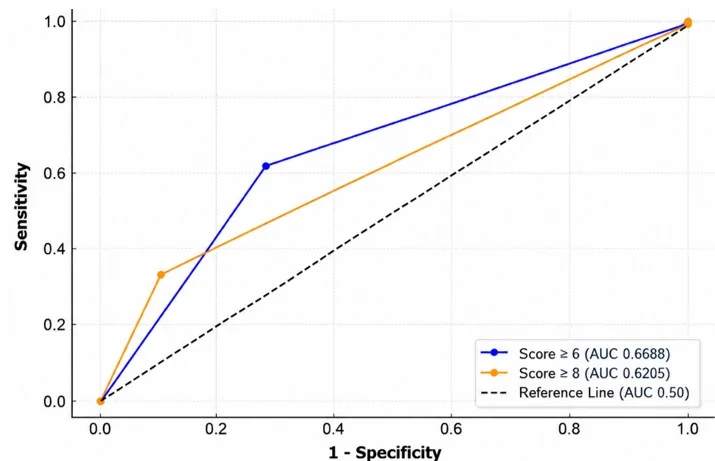
AUC for different LRINEC score cutoff values

## Discussion

During the 6-year study period, 32 cases of NF were identified among 1,546 patients hospitalized with SSTIs at the participating institutions, corresponding to an NF frequency of 2.06% in the SSTI population evaluated. However, among the subgroup of 256 patients with systemic inflammatory response syndrome (suspected cases of NF), 32 cases were confirmed, corresponding to a frequency of 12.5%.

The present study provides pioneering evidence regarding the diagnostic performance of the LRINEC score for assessing the risk of NF in a Latin American population. The mean LRINEC score was 6 points among patients with confirmed NF. Furthermore, an LRINEC score ≥6 yielded a sensitivity of 62.07% and a specificity of 71.7% for the diagnosis of NF, whereas a score ≥8 yielded a sensitivity of 34.48% and a specificity of 89.62%. These findings are particularly relevant because this is the first study conducted in Colombia to evaluate the diagnostic performance of this tool in a population with SSTIs.

The diagnostic performance observed differed from that reported in the original validation study, which included a larger number of patients with NF than the present cohort^6^. Furthermore, the diagnostic performance of the LRINEC score has been shown to vary according to study design and the population evaluated^15^. Compared with other studies conducted in Latin American populations, these findings are consistent with those reported by Ferrer Lozano et al.^11^ in 2019, as well as with findings from retrospective studies such as that conducted by Breidung et al.^16^ at a tertiary care center in Germany. That study included 125 patients with NF and reported a sensitivity of 59% and specificity of 82% for an LRINEC cutoff score ≥6. These findings contrast with those of prospective studies, which have reported higher sensitivity and specificity. For example, a prospective study conducted at three hospitals in India and published in 2020 included 36 patients with NF and reported a sensitivity of 87.8% and specificity of 100% for an LRINEC score ≥88^8^.

The retrospective design of the present study may explain the lower diagnostic performance observed compared with the original validation study. However, when compared with studies conducted in Latin American populations, such as a single-center study conducted in Cuba in 2018 that included 28 cases of NF and reported an AUC of 0.67, a sensitivity of 66%, and specificity of 75%, the diagnostic performance observed was similar to that found in the present study^11^.

When used in isolation and without appropriate clinical context, the LRINEC score has limited diagnostic utility for differentiating NF from other soft tissue infections due to its low sensitivity. Nevertheless, in the study population, it retained adequate specificity and negative predictive value (NPV). Accordingly, an LRINEC score lower than 6 points may help rule out NF, with an NPV of 87%. Systematic reviews and meta-analyses, such as that by Tarricone et al., which included 12 studies conducted primarily in developed countries, have highlighted the utility of the LRINEC score for ruling out NF because of its high NPV, which may reach 89%, despite its limited sensitivity^17^. Other systematic reviews have also reported high specificity and an adequate NPV^18^. However, validation studies conducted in tropical countries have reported limited sensitivity, although these studies have primarily used retrospective designs^19^. This limitation may result in NF being incorrectly ruled out in affected patients when the LRINEC score is used alone. Therefore, the LRINEC score should be used to guide clinical decision-making and the use of additional diagnostic tests in conjunction with clinical judgment. Given its low sensitivity, the findings of the present study are consistent with those reported by other authors: an LRINEC score lower than 6 may help rule out the possibility of NF; however, scores greater than 6 or 8 require complementary diagnostic testing because of the limited sensitivity of the score^20^.

The present study showed that the frequency of NF among patients hospitalized with SSTIs was 2%. Other authors have reported NF in 5.1% to 6.1% of patients with lower-extremity cellulitis^21^. No deaths occurred in the group without NF, whereas in-hospital mortality among patients with NF was 13. This finding is consistent with reports from other populations showing a progressive decline in mortality^22^, lA nationwide study conducted in the United States that included 4,178 NF cases between 2010 and 2014 reported a mortality rate of 12.6%^23^. Similarly, a mortality rate of 11.8% was reported in a study involving a low-income population^24^.

Cases of NF occurred in a population with a mean age of 56 years and were more frequent among men, findings similar to those reported in other populations^25^,^26^. Furthermore, patients with NF in the present study had a higher frequency of chronic comorbidities. Diabetes mellitus was present in 52% of patients, hypertension in 41%, and chronic kidney disease in 20%. Studies conducted in other populations have likewise identified diabetes mellitus as the most common comorbidity among patients with NF^27^.

Although the clinical presentation of NF varies according to the causative microorganism, anatomical site, and depth of infection^28^, severe pain out of proportion to physical findings and extending beyond the margins of inflammation is considered a clinically useful indicator^15^. In the present cohort, pain severity was a clinical characteristic that differed significantly in patients with NF.

Previous studies conducted in other populations have reported fever in 44% of patients with NF, tachycardia in 59%, hypotension in 21%, and tachypnea in 26%, with considerable variability depending on the clinical and epidemiological context^29^–^31^. In the present population, 38% of patients with NF presented with fever, highlighting the need for an integrated clinical assessment that extends beyond a single clinical sign and incorporates the patient’s overall clinical and symptomatic status^32^.

**Strengths and Limitations**

The present study is novel because it is the first study conducted in a Colombian population to describe the clinical and demographic characteristics of patients with NF. It was conducted at two tertiary care university hospitals serving as referral centers. The proposed objectives were achieved, demonstrating the diagnostic performance and validity of the LRINEC score in the Colombian population. The results were similar to those reported in other retrospective studies.

Among the study limitations is its retrospective design. Data quality depended on the accuracy, completeness, and standardization of the medical records. The number of incomplete records led to the exclusion from statistical analysis of 3 suspected cases with confirmed NF and 98 suspected cases in which NF was ruled out. Furthermore, because more than 10% of the records had incomplete data, imputation could not be performed as initially proposed in the study protocol. The retrospective nature of the study and the exclusion of patients with incomplete records should be considered because of the potential for selection bias, which may affect the precision of the LRINEC score and the generalizability of the findings to the broader population. These limitations underscore the need to strengthen clinical data recording systems, improve data standardization, and promote prospective study designs in future research.

## Conclusions

In this study of patients hospitalized with SSTIs in Colombia, NF had a low but clinically relevant frequency, and its associated mortality underscores the importance of early diagnosis. The LRINEC score demonstrated limited diagnostic performance, with greater utility for reducing the probability of NF when the score is low; however, when used alone, it is insufficient as a confirmatory diagnostic tool.

These findings reinforce the need to integrate the LRINEC score with clinical judgment, comprehensive patient assessment, and additional diagnostic tests, particularly when the clinical presentation is atypical. The methodological limitations inherent to the retrospective study design may have influenced the observed diagnostic performance. Prospective multicenter studies are therefore needed to provide a more valid estimate of the true utility of the LRINEC score and to explore modifications or models that may improve its discriminatory capacity.
